# Epigenetic Treatment of Myelodysplastic Syndromes and Acute Myeloid Leukemias

**DOI:** 10.2174/092986708784534947

**Published:** 2008-06

**Authors:** Giuseppe Leone, Francesco D’Alò, Giuseppe Zardo, Maria Teresa Voso, Clara Nervi

**Affiliations:** 1 Istituto di Ematologia, Università Cattolica del Sacro Cuore, Rome, Italy; 2 Dipartimenti di Biotecnologie Cellulari ed Ematologia e di; 3 Istologia ed Embriologia Medica, Università di Roma “La Sapienza”; # San Raffaele Bio-medical Park Foundation, Rome, Italy

**Keywords:** MDS, AML, HDAC inhibitors, hypomethylating agents, epigenetics.

## Abstract

Epigenetic mechanisms affecting chromatin structure contribute to regulate gene expression and assure the inheritance of information, which are essential for the proper expression of key regulatory genes in healthy cells, tissues and organs. In the medical field, an increasing body of evidence indicates that altered gene expression or de-regulated gene function lead to disease. Cancer cells also suffer a profound change in the genomic methylation patterns and chromatin status. Aberrant DNA methylation patterns, changes in chromatin structure and in gene expression are common in all kind of tumor types. However, studies on leukemias have provided paradigmatic examples for the functional implications of the epigenetic alterations in cancer development and progression as well as their relevance for therapeutical targeting.

## INTRODUCTION

**Fig. (1):**
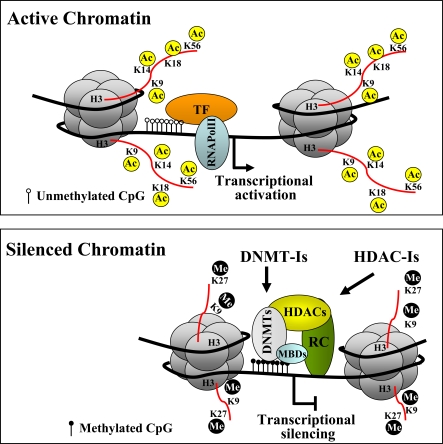
Schematic representation of epigenetic modifications leading to gene transcriptional regulation. Epigenetic modifications of the histone H3 tails, Lys9 (K9), Lys14 (K14), Lys18 (K18), Lys56 (K56), Lys27 (K27) are indicated. Ac: acetylation; Me: methylation. White and black circles indicate the un-methylated and methylated CpG dinucleotides, respectively. TF: transcriptional factors; RNAPolII: RNA polymerase II; DNMTs: DNA methyltransferases; HDACs: Histone deacetylases; MBDs: Methyl-CpG binding protein; RC: transcriptional repressive complex. Arrows indicate the targeting of specific chromatin remodeling enzyme activities by DNA methyltransferase inhibitors (DNMT-Is) and histone deacetylase inhibitors (HDAC-Is).

Epigenetic mechanisms such as DNA methylation, post-translational modifications of histone proteins and remodeling of nucleosomes affect chromatin structure and contribute to define heritable changes in gene expression. In healthy cells, tissues and organs, a proper chromatin structure is indeed essential for the cell and tissue specific control of gene expression and for the recognition and actual accessibility of specific DNA sequences to chromatin-associated proteins, including transcription factors [1]. The nucleosome is the basic unit of chromatin, which consists of 146 bp of DNA wrapped around an octamer of core histone proteins: histone 2A (H2A), histone 2B (H2B), histone 3 (H3) and histone 4 (H4). The dynamic modulation of the chromatin structure is mainly caused by the activity of different chromatin remodeling enzymes including DNA methyl-transferases (DNMTs), proteins binding methylated DNA (MBDs), histone acetyl-transferases (HATs), histone de-acetylases (HDACs), histone methyl-transferases (HMTs) and histone de-methylases (HDTs), which generate a particular array of marks on DNA and histones. These enzymes cause chromatin modifications by affecting: i) the methylation status of cytosine in the DNA CpG dinucleotide (DNMTs); ii) the amino terminal tails of the core histones that protrude through the DNA and are exposed on the nucleosome surface, including, acetylation and deacetylation of lysines, methylation and demethylation of lysine and arginines (HAT, HDACs, HMTs, HTDs), phosphorylation of serines and threonines and others; iii) the adenosine triphosphate-dependent SWI/SNF complex [1-3]. Specific molecular modifications on CpGs and nucleosomal histones affect the higher-order chromatin architecture and function by changing the interaction of histones with DNA or the contact between different histones in adjacent nucleosomes. This allows or denies the accessibility of the transcriptional machinery and of transcription factors to their specific sites on gene promoter regions. This activates or silences the transcription and the expression of genes including those involved in cell determination and tissue specificity (Fig. **1**).

Deregulation of epigenetic mechanisms of gene expression may be as relevant as genetic alterations for the development and progression of cancer and leukemia. Epigenetic deregulation can also cooperate with genetic alteration in tumor and leukemia establishment and progression. Indeed, dysfunctional epigenetic mechanisms controlling gene expression, have acquired great importance as oncogenic factors per se and are considered common marks for tumor cells [4, 5].

Epigenetic transcriptional silencing of genes required for proliferation and differentiation of the hematopoietic cell are likely to contribute to the leukemogenic event underlying myelodysplastic syndromes (MDS) and acute myeloid leukemias (AML). In these diseases, epigenome-modifying enzymes such as HDACs, HATs and DNMTs contribute to transcriptional de-regulation as the consequence of their aberrant recruitment or function at specific target gene promoter sites. In this view, altered DNA methylation patterns and histone marks are not only of importance to our understanding of the molecular pathogenesis of these diseases but may also serve as novel markers for their diagnosis, prognosis, and prediction of response to therapy [6, 7].

Moreover, the potential reversibility of the DNA and chromatin modifications makes chromatin remodeling enzymes attractive targets for therapeutic intervention, opening up new horizon for the treatment of MDS and AML. Two classes of epigenetic drugs are today available in clinical settings, which act through the inhibition of the enzymatic activities responsible for epigenetic transcriptional silencing, the DNMTs and the HDACs, respectively. Re-expression of silenced oncosuppressor genes represents the final aim of epigenetic therapy, but it is not excluded that these drugs may also act through different mechanisms.

### Epigenetic Changes in Normal and Neoplastic Cells

The methylation of cytosine of CpG dinucleotide, within DNA regions of 0.2-5 Kb, heavily enriched for CpG dinucleotides (known as CpG islands), mainly located in promoter regions, is a major epigenetic mechanism of transcriptional control, which is dysregulated in human cancer. In normal cells, cytosines within CpG islands are usually unmethylated, whereas CpG dinucleotides, mainly localized in repetitive, centromeric regions, are methylated. The lack of methylation in promoter-associated CpG islands allows gene transcription to occur by providing both the formation of an active chromatin status and the binding of appropriate transcription factors [6]. Methylation of promoter CpG islands is associated with an inactive chromatin structure and transcriptional silencing of the associated gene [6, 8, 9].

CpG methylation is catalysed by a family of enzymes known as DNA methyltransferases (DNMTs) that add a methyl group to the carbon 5’ position of the cytosine ring in the palindrome CpG dinucleotide [10, 11]. DNMT-1 is a maintenance methylase that recognizes and methylates hemi-methylated CpG dinucleotides during DNA replication allowing the propagation and conservation of DNA methylation patterns through the future generations [12]. DNMT-3a and -3b are *de novo* methylase and methylate unmethylated CpG dinucleotides [13].

DNA methylation patterns are established during the embryogenesis and must be maintained unaltered during adult life to guarantee not only transcriptional silencing, a condensed chromatin structure and chromosome X inactivation in women, but also genomic stability through suppression of homologous recombination between repetitive sequences [1]. Looking at development, it has been shown that most germline-specific genes are later on methylated in somatic cells, suggesting additional functional selection, during differentiation [14].

In cancer cells aberrant promoter hypermethylation coexists together with global hypomethylation [7, 15]. The hypomethylation of pericentromeric and centromeric regions, repetitive elements and integrated/silenced viral sequences exerts the oncogenic effect through reactivation of silenced sequences and of oncogenes [16, 17]. On the other hand, aberrant hypermethylation of promoter CpG islands leads to transcriptional silencing of known or candidate tumour suppressor genes [6, 8, 9]. The frequency of this process, the variety of genes involved, and the large repertoire of cancers shown to harbour dense methylated promoter CpG islands all reflect the critical role of this epigenetic mechanism in cancer initiation and progression. Some genes have been shown to be hypermethylated in many tumour types, but in general, the pattern of genes hypermethylated in cancer cells is tissue specific and not random [7]. Many fundamental components of key cellular pathways are inactivated in human cancer by hypermethylation including: DNA repair (MLH1, MGMT, BRCA1), cell cycle (p16INK4a, p15INK4b, p14Arf), cell invasion and adherence (E-cadherin, APC, CDH13, VHL), apoptosis (DAPK1, caspase 8), detoxification (GSTP1) and hormonal response (retinoic acid receptor β2 and estrogen receptor). The deregulation of such pathways is likely to confer a survival advantage to the affected cell and thus to contribute to the step-wise progression towards carcinogenesis [7, 8].

However, the effects of CpG island promoter methylation on transcription depend not only on DNA methylation, but also on additional epigenetic events such as modification of histone tails and recruitment of methylated DNA binding proteins [18]. Chromatin remodeling involves proteins with high affinity for methylated CpGs, known as methyl CpG binding proteins MeCP2, MBD1, MBD4 and Kaiso, which mediate the inhibitory effect of CpG island methylation on gene expression acting as transcriptional repressors. Methyl CpG binding proteins are also often part of large co-repressor complexes comprising, NuRD, NoRC, N-Cor, mSin3A and SWI-SNF [19-22]. These repressor activities also recruit HDACs and HMTs on methylated targeted promoter sequences. The consequent post-translational modification of histone tails induced by these enzymes determines a silenced transcriptional status of chromatin [1, 18, 19, 23].

*In vitro* studies suggest that HAT and HDACs can target several ε amino groups of evolutionary conserved lysine residues present on N-terminal region of nucleosomal histone H3 and H4 [24]. The acetylation of lysine residues on the N-terminus of histones by HATs has the most potential to unfold chromatin and is generally associated with activation of transcription. Most HATs can also acetylate proteins other than histones [25-27].

In contrast, the HDACs induced de-acetylation of lysine residues on histones (and potentially on other proteins), has the potential to compact chromatin resulting in transcriptional gene repression [28-30]. Mammalian HDACs are grouped into four families: the class I, II, III and IV of HDACs. Class I includes HDACs 1, 2, 3 and 8, which are homologs of the yeast RPD3 protein, whereas HDACs 4, 5, 6, 7, 9 and 10 that are related to the yeast Hda 1 protein form class II. HDAC11 is the unique member of HDAC class IV. HDAC class III includes recently identified mammalian homologs of the yeast Sir2 protein. In cells, HDACs are present as subunits of multiprotein complexes. The cellular sublocalization of HDAC class I is constitutively nuclear and dynamically affects gene regulation, while HDAC class II translocates from the cytoplasm to the nucleus in response to external stimuli. HDAC class I and II interact with adaptor protein like mSin3A and transcriptional co-repressors N-CoR and SMRT to repress gene transcription [31].

HMTs methylate specific lysine or arginine residues on nucleosomal histone H3, which in the case of lysine 9 (K9) methylation allows the recruitment of the heterochromatin protein 1 (HP1) stabilizing an inactive condensed chromatin [3]. Histone tail modifications and DNA methylation gather to assemble chromatin structure, which dynamically shift from a transcriptional permissive state to a transcriptional inactive state and viceversa [32]. Hypermethylated and silenced genes in cancer are known to have key histone modifications in their promoter regions (i.g. the deacetylation of K9, demethylation of K4 and methylation of K9 and K27 on histone H3) [28, 34, 35].

### Acute Myeloid Leukaemia and Myelodysplastic Syndromes

Whereas aberrant DNA methylation patterns, changes in chromatin structure and in gene expression are common in different tumor types, studies on leukemias have provided paradigmatic examples for the functional implications of genetic and epigenetic alterations in cancer development. Several studies have shown that methylation-associated silencing inactivates certain tumour suppressor genes as effectively as mutations and that it may act as one of the cancer predisposing hits described in Knudson’s two-hit hypothesis [9, 36, 37]. Indeed, oncogenic fusion proteins resulting form chromosomal translocations associated to acute myeloid leukemias (i.e. PML/RAR, AML1/ETO), exert their oncogenic effect by an aberrant recruitment of repressor complexes, containing DNMT and HDAC activities, on genes that are relevant for hematopoietic differentiation [31]. Moreover, missense mutations, rearrangements or inactivation by oncogenic proteins of histone acetyltransferase activities (HATs) such as CREB Binding Protein (CBP) or p300 have also been reported in leukemias [38, 39]. Suppression of α-catenin (CTNNA1) expression by both methylation and histone deacetylation has been shown to contribute to the growth advantage in human MDS or AML with del(5q) [40]. Similar to acute promyelocytic leukemia, the differentiation block due to the t(8;21) translocation is the result of the recruitment by AML1/ETO of chromatin remodeling enzymes, leading to epigenetic silencing of the microRNA miR-223 [41].

Many genes, important for most of the carcinogenic pathways, are frequently hypermethylated in MDS and AML (Table **1**). Accordingly, it has been shown that DNMT1 and DNMT3A are overexpressed in MDS and AML samples, when compared to controls [42]. Among proliferation markers, the tumor suppressor gene p15 (CDKN2B), a cyclin dependent kinase inhibitor, target of TGFβ, important for the G1/S phase transition of the cell cycle, has been shown to be hypermethylated in 40-80% of MDS [43]. The pattern of methylation was not different between hematopoietic progenitor cells and differentiated cells [44] and was associated to poor survival [45]. Furthermore in acute myelogenous leukemia, increased p15 methylation levels were found in CD34+ cells of many leukemic patients in haematological remission and was associated with a high relapse rate and significantly reduced relapse-free survival [46].

Gene promoter hypermethylation is not always associated to poor prognosis. Higher levels of methylation in multiple genes (NOR1, NPM2,OLIG2, HINI, SLC26A4) were found in patients with good prognosis AML, while no significant difference in methylation of p15, CDH13 and PGR was found between good and poor prognosis groups [Kroeger *et al.* ASH 2007, Blood 110, Abstract #595]. These data suggest that methylation does not always have the same prognostic significance.

Increased angiogenesis has been shown to be involved in the pathogenesis of MDS, accordingly we found hypermethylation of the anti-angiogenic protein thrombospondin in 6% of MDS, especially those with a intermediate-2/high IPSS score (unpublished observation). Similarly the metastasis suppressor gene E-cadherin (CDH1), which binds to α- and β-catenin, has been shown to be hypermethylated in one third of MDS samples [47].

## PHARMACOLOGY OF HYPOMETHYLATING AGENTS

Epigenetic changes in malignancy are potentially reversible by therapeutic inhibition of DNA methylation or histone deacetylation and the differential methylation of CpG islands is now a target for therapy [48]. The rationale for pharmacological reversion of the methylator phenotype is the reactivation of tumour suppressor gene expression. The hypomethylating agents, azacitidine and decitabine were shown to inhibit DNMT1, leading to induction of expression of silenced genes [48], to cause growth inhibition and modest differentiation of transformed myeloid cell lines and primary leukemic blasts i*n vitro* [49], and to produce clinical responses in AML and MDS patients *in vivo*. Both azacitidine and decitabine are the first drugs specifically approved by the USA Food and Drug Administration (FDA) for the treatment of MDS. Such agents offer a novel therapeutic approach, which is less intensive than standard chemotherapy. Clinical trials using DNMT inhibitors alone or in combination with HDAC inhibitors recently yielded promising results in patients with other haematological malignancies [7, 48].

### General Features and Mechanisms of Action of Hypomethylating Agents

Azanucleotides are cytidine analogues modified in position 5 of the pyrimidine ring with the presence of a nitrogen atom substituting a carbon. Azacitidine (5-azacytidine, VidazaTM, Pharmion Corporation, Boulder, CO, USA) and decitabine (5-aza-2’- deoxycytydine, Dacogen™, MGI PHARMA, Inc., Bloomington, MN, USA), first synthesized in the early 1960s, by Sorm and co-workers [50, 51], are the best-charactherized drugs belonging to this class. At high doses, azacitidine and decitabine are cytotoxic, like other cytidine analogues such as cytarabine. However it was later found that the ability of these drugs at low doses to inhibit DNMT1 and cause DNA hypomethylation, thereby restoring normal function to genes that have a key role in the control of cellular differentiation and proliferation [52-54]. Indeed, concentration of azacitidine and decitabine required for maximum inhibition of DNA methylation *in vitro* were not shown to suppress DNA synthesis [55]. Several other compunds were shown to have demethylating activity, including 5-fluoro-2’-deoxycytidine, procaine, procainamide, hydralazine and (-)-epigallocatechin-3-gallate, but all these drugs have been so far less effective than the first azanucleotides [56].

**Fig. (2):**
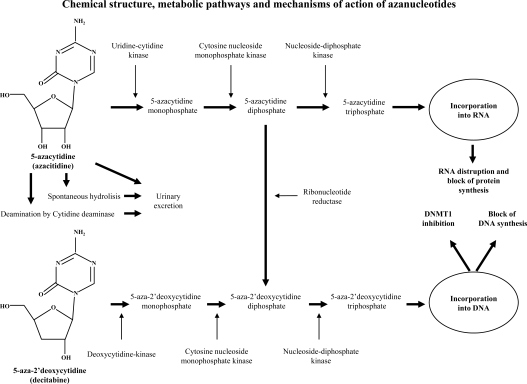
Intracellular methabolic pathways of azacitidine and decitabine. After cellular uptake, both drugs require phosphorilation to triphosphate derivates to be incorporated respectively into newly synthesized RNA and DNA. 5-azacytidine diphosphate is also reduced by ribonucletide reductase to 5-aza-2’deoxycytidine diphosphate which is furthermore phosphorilated and incorporated into DNA. Then, the 5-azacytosine rings bind covalently the DNMT1 and the resulting adducts are excised from DNA, ubiquitinilated and targeted to proteosome for degradation, inducing the lost of methylation in one of the DNA daughter molecules and re-expression of silenced genes. Azacitidine also induces RNA degradation and inhibition of protein synthesis while both drugs at high doses block the DNA synthesis.

Both azanucleotides are pro-drugs: following uptake into the cell by nucleoside-specific transport mechanisms, azacitidine and decitabine are phosphorylated to monophosphate derivative by uridine-cytidine kinase and deoxycytidine-kinase, respectively, and then to diphosphate and triphosphate derivatives by pyrimidine monophosphate and diphosphate kinases. 5-azacytidine triphosphate is incorporated into RNA, disrupts nuclear and cytoplasmatic RNA and inhibits protein synthesis, while 5-aza-2’deoxycytidine triphosphate is incorporated into replicating DNA, and inhibits DNA methylation at low doses and DNA synthesis at high doses. Moreover, 5-azacytidine diphosphate is reduced by ribonucleotide reductase to 5-aza-2’deoxycytidine diphosphate, which is then phosphorylated to 5-aza-2’deoxycytidine triphosphate and incorporated into DNA (Fig. **2**). As a result, while decitabine derived triphosphate azanucleotides are incorporated into DNA only, azacitidine derived triphosphate azanucleotides are incorporated both into DNA and RNA [55, 57]. After integration into DNA of replicating cells, the 5-azacytosine ring covalently bind the mantainance methyltransferase DNMT1 that is then polyubiquitinated and targeted for degradation in the proteosome [58]. In this way, DNMT1 is no more available to remethylate hemi-methylated sites created during DNA replication, and methylation is passively lost in one of the daughter DNA molecules during cell division, inducing re-expression of gene silenced by promoter hypermethylation [59]. Since in cancer cell DNA methylation usually affects tumor suppressor genes which are genetically intact, their reactivation after demethylation completely restores their normal functions. Demethylating agents were also shown to induce in tumor cells cancer/ testis and viral antigens, that may represent a target for humoral and CD8+ T immune response against tumor, providing opportunities for immunotherapeutic targeting [60, 61].

While the demethylating effect depends on incorporation of derived deoxy-azanucleotides into DNA, azacitidine-induced cytotoxicity is mainly due to incorporation into RNA in the cell cycle phase G1 at low drug concentrations and to the incorporation into both RNA and DNA in the G1 and S phases of the cell cycle at higher concentrations of azacitidine [62]. On the other end, decitabine is a phase-S specific drug because its activity is exclusively related to incorporation into DNA of replicating cells.

Major mechanisms of resistance to azanucleotides are represented by increase in cytidine deaminase, the enzyme that inactives both drugs [63]. Zebularine (1-(Beta-d-ribofuranosyl)-1,2-dihydropyrimidin-2-one), is an acid stable and oral available nucleoside analogue that endows with unique biologic properties as a potent inhibitor of both cytidine deaminase and DNMTs, due to the lack of the exocyclic 4-amino group in the pyrimidine ring [64]. Although zebularine possesses an independent antineoplastic activity, this drug was shown to synergize with decitabine in reactivating methylated genes and inhibiting proliferation of leukemic cell lines *in vitro*, by blocking decitabine inactivation through cytidine deaminase [65]. This combination will offer new options to increase the DNMT inhibitor activity in patients.

Another attempted approach to inhibit the DNMTs was represented by a phosphorothioate antisense oligodeoxynucleotide directed against the 3' untranslated region of the DNMT1 enzyme mRNA (MG98). This agent was able to inhibit DNMT1 expression without effecting DNMT3, causing demethylation with reexpression of silenced genes in cancer cell lines and tumor growth inhibition in mouse models. However, an unfavourable toxicity profile and no clinical responses were observed in patients with solid tumors treated by MG98 in phase I and II clinical trials [66, 67].

### Pharmacological Profile of Azacitidine

Azacitidine can be administered intravenously (i.v.) or subcutaneously (s.c.). Maximum plasma concentrations (Cmax) occur 30 minutes after s.c. administration and 11 minutes after a 10 minutes i.v. infusion. The mean plasma concentration following i.v. infusion is approximately 4-fold higher than that following s.c. administration. The bioavailability after s.c. administration is 89% of that after i.v. infusion. The plasma half-life is approximately 22 minutes after i.v. infusion and 41 minutes after s.c. administration. The drug is widely distributed in tissues with a mean distribution volume of 76 liters. Azacitidine is very unstable in aqueous solutions, and after administration it undergoes rapid deamination by cytidine deaminase with subsequent degradation. Azacitidine and its metabolites are mainly excreted by the urinary tract with a mean elimination half-life of about 4 hours [68]. There are no data about the interaction of azacitidine with other drugs.

The main target organs of toxicity after treatment with azacitidine are the bone marrow and the gastrointestinal tract. Common side effects reported by patients after azacitidine treatment include nausea, vomiting, diarrhea, constipation, anorexia, injection site events, arthralgia, cough, dyspnea, headache, weakness, dizziness, and insomnia. Hematological toxicity includes thrombocytopenia and neutropenia, leading to bleeding and to an increased infection risk. In the CALGB 9221 trial, grade 3 and 4 granulocytopenia occurred in 58% and thrombocytopenia in 52% of patients receiving azacitidine. Toxicity was transient and patients usually recovered in time for the next treatment cycle. The highest proportion of myelosuppression occurred during the first two cycles of therapy and decreased in subsequent cycles [68, 69].

A peculiar concern about azacitidine mechanim of action regards the hypothetical secondary tumorigenesis related to the drug-induced global DNA hypomethylation. Mice carrying hypomorphic DNMT1 with reduced expression to 10% of wild type levels, show genome-wide DNA hypomethylation and develop aggressive T-cell lympomas [70]. Moreover studies *in vitro* and in rodents have shown that azacitidine is mutagenic, embryotoxic, teratogenic and carcinogenic, as other pyrimidine analogs [71, 72] and azacitidine treated rats developed a variety of tumor types, including acute leukemia, malignant reticuloendotheliosis, tumors of testis, skin, bronchus, and often multiple tumors [73]. So far, there are no published reports about secondary malignancies developing following azacitidine treatment in humans, but a careful and longer follow-up and the growing number of treated patients will clarify this issue.

An oral formulation of azacitidine in now available by Pharmion Corporation (Boulder, CO, USA) and a Phase 1 clinical trial is ongoing to assess safety, tolerability, bioavailability and pharmacokinetics of escalating single doses in patients with MDS and AML [ClinicalTrials.gov Identifier: NCT00528983]. A pharmacokinetic study carried out in dogs treated with oral azacitidine demonstrated rapid absorption with absolute bioavailability of 67% and plasma concentrations similar to those obtained with parental doses [Ward *et al.* ASCO2007, Abstract #7084].

### Pharmacological Profile of Decitabine

Decitabine is available for i.v. administration. Oral administration is not optimal due to the rapid decomposition in acid. There is an excellent distribution of decitabine in the body fluids after i.v. injection with rapid equilibration between the extracellular and intracellular compartments [74]. Decitabine crosses the blood-brain barrier and during a continuous i.v. infusion the level of this analogue in the cerebral spinal fluid is about half the plasma level [75]. Clearing of meningeal leukemia was observed in a patient treated with decitabine continuous intravenous infusion [76].

In the most common schedule used for MDS and AML, decitabine was administered at a dose of 15 mg/m^2^ as a 3 h i.v. infusion every 8 h for three consecutive days of a 6-week cycle, with a total dose per cycle of 135 mg/m^2^ [77, 78], but several other schedules of administration have been studied [79]. After a single 15 mg/m^2^ 3 h i.v. infusion, C_max_ is 64.8–77.0 ng/ml and is generally reached at the end of infusion. The mean area under the plasma concentration-time curve (AUC) range from 152 to 163 ng h/ml. Steady state in plasma is reached by the end of 3 h infusion. The terminal phase elimination half life (t_½_) is approximally 35 minutes and no systematic accumulation of the drug occurs during repeated dosing due to the short t_½_. The mean total body clearance values were 125-132 l/h per m^2^, indicating that both hepatic and extrahepatic metabolism are involved in elimination of decitabine. Cytidine deaminase plays the major role in the deactivation process by deamination of decitabine. The mean values of distribution volume at steady state ranged from 62.7 to 89.2 l/m^2^ [74, 80].

Decitabine is generally well tolerated and has a favorable toxicity profile. Myelotoxicity is the most important adverse effect observed in patients who received decitabine therapy. Most episodes of febrile neutropenia and sepsis occurred in MDS patients during the first or second cycle of therapy, when neutropenia due to both therapy and/or disease was present. Less common side effects include nausea, vomiting, anorexia, mucositis, alopecia, liver toxicity, renal failure, atrial fibrillation, seizures, sleep disorder, deafness [74, 78].

Chromosomal aberrations and single-strand breaks in DNA have been detected in cells after treatment with decitabine, although the single-strand DNA breaks may represent a repair mechanism to remove DNMT adducts containing 5-azacytosine from DNA [81, 82]. As for azacitidine, concerns exist about a possible carcinogenic effect of drug-induced global genome hypometylation. Nevertheless, it was shown that pharmacologically induced demethylation is only transient with restoration of normal methylation levels in few weeks after stopping therapy [83], and decitabine has shown neither significant mutagenic activity *in vitro* nor carcinogenic properties *in* *vivo* [74]. Moreover mice with a genetic predisposition to develop colon or lung cancer showed a marked reduction in tumor formation when treated with decitabine, suggesting some chemopreventive potential [84].

## PHARMACOLOGY OF HDAC INHIBITORS

### General Features and Mechanisms of Action of HDAC Inhibitors

After the first observations about the antitumoral activity of trichostatin A and trapoxin, whose HDAC inhibiting activity was later discovered [85, 86], a relatively wide range of compounds deriving from both natural sources and from synthetic routes, have been identified to be able to inhibit the activity of class I, class II and class IV HDACs [31, 87]. Most of these HDAC inhibitors (HDACIs) work equally well against all HDACs, but some of them preferentially inhibit class I versus class II HDACs. Howerver, despite a different HDAC isoenzyme inhibition profile and pharmacological properties, all HDACIs show similar patterns of cellular response, and there is no definitive evidence that distinct HDACs have a defined role in cancer [88].

The active site of all HDAC consists of a narrow tubular pocket with a zinc atom inside. Despite the variety of their structural characteristics, HDAC inhibitors can be broadly characterized by a common “pharmacophore” model which consists in a surface domain (also named “CAP”), which is able to interact with the rim space at the entrance of the catalytic tunnel of the enzyme, linked to a hydrophobic spacer through a polar connection unit. At the end of the hydrophobic spacer, a zinc-binding domain assures the inhibition of enzyme activity. Since structural features around the active site are well conserved across all the HDACs, except for the rim of the catalytic pocket, changes of CAP and/or connection unit might justify different potency and selectivity of HDAC inhibitors [89, 90].

After HDAC inhibition with the resulting hyperacetylation of lysine residues in the histone tails, chromatin structure shifts to a transcriptionally active state, with re-expression of silenced genes. It is thought that from 5 to 20% of all known genes are affected by HDACIs [91-93]. However, acetylation works together with other post-translational modifications, and blocking deacetylation might have very different outcomes depending on the previous chromatin state. Additionally, since a growing number of non-histone proteins are recognized being HDAC substrates, it is likely that part of HDACI effects on cellular profile might be independent of chromatin state modification. Indeed, there are multiple mechanisms by which HDACIs can influence the expression of a protein, other than the level of mRNA production, such as modifying stability of the protein by altering chaperone protein function or preventing ubiquitinilation and proteosome degradation. Moreover HDACIs may interfere with the subcellular localization, DNA-binding activity, protein-protein interaction of several non histone proteins, such as transcription factors or signal transducers, by increasing their acetylation [31].

HDACIs induce, to a variable extent, growth arrest, differentiation or apoptosis *in vitro* and *in vivo*. Growth arrest is usually induced at low doses, while apoptosis occurs at higher doses. HDACIs induce cytoxicity in a broad selection of tumor and normal cell lines, both in cycling and non-proliferating cells, although higher drugs concentration are required to kill arrested cells [31]. Normal cells usually show a strikingly reduced sensitivity to HDACIs treatment, hinting at potentially large differences in the acetylome in normal versus tumour cells [94].

HDACI-induced growth arrest is tightly linked to the induction of p21, through both hyper-acetylation of chromatin at the CDKN1A (which encodes p21) promoter and transcriptional activation of CDKN1A [95, 96]. HDACIs also induce cell death through caspase-dependent and caspase independent pathways, and through the accumulation of reactive oxygen species (ROS) [97, 98]. Expression of thioredoxin, a ROS scavenger, in normal cells but not in transformed cells might in part justify the reduced sensitivity of normal cells to HDACIs treatment [94]. It was also highlighted the role of BAX in HDACIs induced apoptosis, since BAX-/- murine fibroblast were shown to be resistant to HDACIs. It was demonstrated that the DNA damage associated protein, Ku70, in its deacetylated form (maintained by several HDACs), keeps BAX away from the mitochondrion and protects cells from apoptosis. Treating cells with HDACIs induces hyperacetylation of Ku70 through the HATs, leading to dissociation of the complex and translocation of BAX to the mitochondria with activation of the intrinsic pathway of apoptosis [99, 100].

Since acetylation of core histones has been correlated with chromatin assembly, DNA repair and recombination, HDACIs were shown to inhibit also DNA repair responses in cell lines, which might increase the sensitivity of tumour cells to chemotherapy and radiotherapy by leading to increased DNA damage by these treatments [101, 102].

**Fig. (3):**
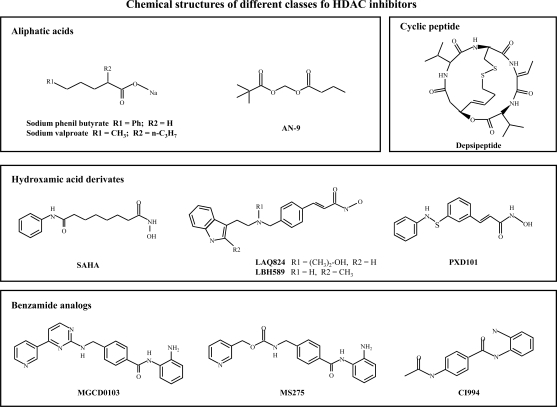
Chemical structure of different classes of HDAC inhibitors entered in clinical trials.

Several thousands of molecules, screened by *in vitro* assays, have been so far shown to have HDAC inhibitory activity. However HDACIs entered in clinical trials can be divided into four chemical classes including short chain fatty acids, hydroxamic acid derivatives, benzamides and cyclic peptides [31] (Fig. **3**).

For the aim of this review, we will discuss only the pharmacological properties of HDACIs entered in clinical trials for the treatment of MDS and AML.

### Short Chain Fatty Acids

These drugs inhibit both class I and IIa HDACs, but usually show low potency due to the inability to make significant contact with the catalytic pocket of HDACs [103] (Table **2**).

Sodium butyrate is the prototype of this class of compounds, *in vitro* induces growth arrest and differentiation of human leukemia cells at millimolar concentrations, but its clinical development has been hampered by its short half-life and difficulty in achieving millimolar levels *in vivo* [104].

Phenylbutyrate is a aromatic fatty acid, able to induce hyperacetylation of histones H3 and H4 and growth arrest, differentiation and apoptosis of AML cell lines and primary leukemic cells. It has been effectively used to induce fetal erythropoiesis in patients with sickle cell anemia and β-thalassemia [105]. The aromatic ring does not contribute to the antitumor activity, as butyric acid is of equal or greater potency at producing these biological changes, while shortening of the fatty acid carbon chain length, as demonstrated with phenylacetate, significantly diminished drug potency [106]. After administration phenylbutyrate is metabolized to phenylacetate, then to phenylacetylglutamine and eliminated by urine [107]. The maximum tolerated doses, when administered as a 7-day continuous infusion, was 375 mg/kg/day, while higher doses were associated with encephalopathy apparently attributable to accumulation of the metabolite phenylacetate. At the maximum tolerated dose (MTD), median steady state concentration of phenylbutyrate is 0.3 mM, which is less than the ED50 of 1-2 mM required for differentiation and cytostasis *in vitro* but in whitin the concentration range in which phenylbutyrate induces acetylation of histones. Dose-limiting toxicities were mainly represented by neurocortical toxicity, including lethargy, confusion, and slurred speech, which completely disappeared within 24 to 48 h upon cessation of the infusion. Non dose-limiting toxicities were hyperammoniemia, hyperuricemia, hypocalcemia, skin abnormalities and interstitial pneumonia [108, 109].

Pivaloyloxymethyl butyrate (AN-9, Pivanex™, Titan Pharmaceuticals, Inc., South San Francisco, CA, USA) is an acyloxyalkyl ester pro-drug of butyric acid that undergoes to rapid hydrolysis to butyric acid, pivalic acid and formaldehyde, after the cellular uptake. Its anticancer effect is assumed to stem primarily from the inhibition of HDACs by the released butyric acid [110]. AN-9 inhibits cell proliferation of a variety of cancer cell lines and primary human solid tumor cells. In leukemia cell lines, AN-9 has shown differentiating and pro-apoptotic effects [111]. Compared to sodium butyrate, AN-9 is at least 10-fold more potent both *in vitro* and *in vivo*, probably due to its increased permeability across cell membranes, allowing for efficient delivery of butyric acid to subcellular targets [112]. Moreover AN-9 has demonstrated more favorable toxicological and pharmacological properties than sodium butyrate in preclinical studies. In phase I clinical studies in patients with advanced solid malignancies, AN-9 was administered by 6-h i.v. infusion daily for 5 days every 3 weeks at dosages ranging from 0.047 to 3.3 g/m^2^/day. The most common observed toxicities were nausea, vomiting, hepatic transaminase elevation, hyperglycemia, fever, fatigue, anorexia, injection site reaction, diarrhea, and visual complaints [113].

Valproic acid (di-n-propylacetic acid) is a short chain fatty acid used for decades as a anticonvulsant, whose anticancer activity was detected *in vitro* more than 10 years ago. However, it was only in 2001 that its antineoplastic effects were shown to depend on its inhibitory action on HDACs [114, 115]. Valproic acid inhibits class I HDACs (HDAC 1 through 3) and class II HDACs 4, 5 and 7 whereas class II HDACs 6 and 10 are not inhibited [103]. In contrast to other inhibitors, it induces also proteasomal degradation of HDAC2 [116]. Valproic acid induces differentiation of transformed hematopoietic progenitors and primary AML blasts, as well as it reduces tumor growth and metastases in animal studies [117]. Oral bioavailability is nearly 100%, but i.v. administration is possible too. It has a small volume of distribution (0.13–0.19 L/kg), the plasma half-life is from 9 to 18 hours, and its protein binding is from 80% to 95%. Multiple interactions with other drugs have been described both at the level of protein binding and drug metabolism [118]. Generally, valproic acid is well tolerated. Main reported toxicities are neurologic side effects such as sedation, dizziness, and tremor, mild gastrointestinal side effects and hematologic toxicity, including pancytopenia and severe bone marrow hypoplasia. Liver failure and teratogenicity with neural-tube defects have been described [117].

### Hydroxamic Acids

This class includes some of the most potent HDACIs. According to the pharmacophore model, the hydroxamic group chelates the zinc atom at the active site of HDACs, while a hydrophobic cap and an aliphatic side chain respectively, interacts with the edge and fits into the hydrophobic catalytic pocket [89, 90]. Members of this class, with trichostatin A (TSA) as the prototype, are potent unselective inhibitors of both class I and II HDACs (Table **2**).

Suberoylanilide hydroxamic acid (SAHA, vorinostat, Zolinza™, Merck & Co., Inc, Whitehouse Station, N.J., USA) is a second-generation hydroxamate compound, which was shown to induce differentiation, growth arrest, or apoptosis of transformed human cells in culture at micromolar concentrations, and to have antitumor activity in several *in vivo* models of cancer [119]. In HDAC enzyme activity assay, hydroxamates derivatives SAHA and LAQ824/LBH589 induce at nM to μM concentration complete inhibition of HeLa nuclear extract HDAC activity as well as recombinant HDAC1, 3, 6 and 8, although the inhibition of HDAC8 was significantly weaker [88].

Clinical responses to SAHA were observed in phase I trials in both refractory solid and hematological malignancies. Increased accumulation of acetylated histones in tumors, bone marrow and peripheral blood cells was observed during the treatment. SAHA was usually well tolerated both i.v. and orally, with different pharmacokinetics and toxicity profile according to the administration pathway. Major adverse events with the oral formulation include fatigue, diarrhea, anorexia, and dehydration. Myelosuppression and thrombocytopenia are more prominent with SAHA i.v. formulation and in patients with hematological malignancies than in those with solid tumors. Usually, the hematologic toxicities resolve shortly after SAHA stopping. The MTD of SAHA for i.v. administration in patients with hematologic malignancies was 300 mg/m^2^/d for 5 days every 3 weeks, while the MTD for the oral formulation was 400 mg qd and 200 mg bid for continuous daily dosing and 300 mg bid for 3 consecutive days per week dosing. At the steady-state of equivalent doses, the Cmax of the i.v. formulation is nearly four fold greater than the Cmax of the oral formulation, whereas the AUC is nearly 22-fold greater for the oral drug. The mean apparent t_½_ following oral administration (range 91.6 to 127 minutes) is longer than the mean apparent t_½_ following i.v. administration of the oral equivalent doses (range 34.7 to 42.4 minutes). After oral administration the estimated bioavailability of SAHA is 43% and food does not appear to alter substantially the rate or extent of absorption [120, 121]. On June 2006, SAHA received FDA approval for the treatment of advanced cutaneous T-cell-lymphoma (CTCL).

LAQ824 and the more potent analog LBH589 are novel cinnamic acid hydroxamates and pan-HDAC inhibitors, developed by Novartis. As other HDACIs, at nanomolar concentrations they induce growth arrest and apoptosis of a variety of cancer and leukemia cell types, cause hyperacetylation of H3 and H4 histones, increase p21 levels, and induce cell cycle G1 phase accumulation [122]. They have been extensively studied for their ability to induce acetylation and to inhibit the ATP binding and chaperone function of heat shock protein (HSP) 90, promoting the polyubiquitylation and proteasomal degradation of the pro-growth and prosurvival client proteins Bcr-Abl, mutant FLT-3, c-Raf, and AKT in human leukemia cells [123]. In a phase I study, LBH589 was administered i.v. as a 30-minute infusion on days 1 to 7 of a 21-day cycle. LBH589 plasma concentration peaked either at midpoint or at the end of the 0.5-hour infusion, then dropped rapidly within the first 4 hours to reach terminal phase between 4 and 24 hours. The terminal half-live ranged from 8 to 16 hours. Intravenous administration of LBH589 was usually well tolerated at doses <11.5 mg/m^2^ with consistent transient antileukemic and biological effects. Potentially LBH589-related toxicities included asymptomatic QTcF prolongation, nausea, diarrhea, vomiting, hypokalemia, loss of appetite and thrombocytopenia [124].

PXD101 (belinostat) is a novel hydroxamate-type inhibitor of histone deacetylase activity that inhibits histone deacetylase activity in HeLa cell extracts with an IC50 of 27 nM and induces a concentration-dependent increase in acetylation of histone H4 in tumor cell lines. PXD101 is cytotoxic *in vitro* in a number of tumor cell lines with IC50s in the range 0.2–3.4 μM and caused a significant dose-dependent growth delay in nude mice bearing human ovarian and colon tumor xenografts with no significant toxicity [125]. In phase I trial conducted in patients with advanced solid tumors, the most common adverse events were fatigue, nausea, vomiting, dysgeusia, dehydration, anorexia and phlebitis. MTD was 1000 mg/m^2^/day, while oral bioavailability was 33% [87].

### Benzamides

Although structurally diverse, these compounds contain a benzamide moiety and are selective, potent inhibitors of class I HDACs. In HDAC enzyme activity assay, the benzamide analogs CI994, MS275 and MGCD0103 are partial inhibitors of HeLa nuclear extract HDACs activity, as they show full and potent inhibition of HDAC1 and 3 isoenzymes, but no inhibition of HDAC6 occurs and high μM concentrations are requested to inhibit HDAC8 [88] (Table **2**).

MS-275 is an orally available 2-aminophenyl benzamide which exerts antiproliferative effects at micromolar levels against a panoply of human tumor cells *in vitro*, including leukemia cell lines and primary leukemia blasts. It has been shown also to induce TGF-β receptor and trigger the intrinsic pathway of apoptosis [126]. MS-275 induces the accumulation of reactive oxigen species (ROS) in transformed but not normal cells, which was associated with upregulation of thioredoxin [94, 97]. MS-275 was shown to synergize with azacitidine to induce cytotoxicity and apoptosis in AML and ALL cells [127]. Since the half-life of the oral formulation of MS-275 in humans ranges from 39 to 80 hours, once weekly and once every-two weeks schedules were used in phase I clinical trials. The maximum-tolerated dose was 8 mg/m*2* weekly for 4 weeks every 6 weeks. Dose-limiting toxicities included infections and neurologic toxicity manifesting as unsteady gait and somnolence. Other frequent non-DLTs were fatigue, anorexia, nausea, vomiting, hypoalbuminemia, and hypocalcemia [128, 129].

CI-994 (4-acetylamino-N-(2'aminophenyl)-benzamide) is a substituted benzamide derivative, which has broad antitumor activity with higher selectivity toward a variety of solid tumor models compared to leukemia cell lines. Due to its lack of aqueous solubility, it requires oral administration. Following CI-994 administration, inhibition of both histone deacetylation and cellular proliferation at the G1 to S transition phase of the cell cycle were observed [130, 131]. In Phase 1 study conducted in patients with solid tumors, MTD was 8 mg/m^2^/day for 8 weeks repeated after a 2-week drug-free interval. Thrombocytopenia was DLT, while other toxicities included fatigue and gastrointestinal effects [132]. In a pharmacokinetic study on non human primates there was an excellent CSF penetration of CI-994 after i.v. administration [133].

MGCD0103 is a novel, orally bioavailable anilide-based HDACI developed by MethylGene Inc. in collaboration with Pharmion Corporation. This molecule is selective for the class I HDACs, and has been shown to inhibit proliferation of a wide variety of cancer cell lines and to enhance the activity of several different chemotherapeutics. After oral administration t_½_ ranges from 7 to 12 hr, t_max_ is 0.6-1 hr, and C_max_ and AUC increase in a dose-dependent manner. The inhibition of HDAC activity in whole cells extends beyond the plasma half-life of the drug, lasting as long as 24 hours post drug administration. Two-times and three-times weekly schedule were studied in Phase I trials both in solid tumors and hematological malignancies [87]. MGCD0103 has been well tolerated at doses below 80 mg/m*2*/day two times weekly and non-dose limiting toxicities included fatigue, nausea, diarrhea and vomiting [Lancet *et al.* ASCO2007, Abstract #2516]. No drug-associated chemistry or hematology toxicities have been observed to date. On August 2007, the FDA granted orphan drug designation to MGCD0103 for the treatment of Hodgkin's lymphoma.

### Cyclic Peptides

This class of HDACIs includes both epoxyketone-containing and non-epoxyketone-containing tetrapeptides. The most characterized drugs belonging to this class is Despipepdide (FK228, Romidepsin), a non-epoxyketone-containing bicyclic tetrapeptide HDACI. Depsipeptide is a natural product isolated from Chromobacterium violaceum with antiproliferative activity in a wide variety of murine and human tumor cell lines both *in vitro* and *in* *vivo* [87]. Depsipeptide is a pro-drug and the active moiety is a sulfhydryl group acting as the Zn+^2^-chelator. It is a more selective inhibitor of the class I HDACs, preferentially blocking HDACs 1 and 2 versus HDACs 4 and 6 [134] (Table **2**). As it is a natural product tetrapeptide, depsipeptide is a substrate of MDR-1 and no cross-resistance has been observed with other cytotoxic agents [87].

There have been performed multiple Phases I and II trials with depsipeptide, both in solid tumors and in hematological malignancies. After a 4 h i.v. infusion, all the plasma concentration time profiles fit a two-compartment model. Generally, it is well tolerated with favorable toxicity profile. Common side effects consist of fatigue, nausea, vomiting, and transient thrombocytopenia and neutropenia. Several ECG findings have been described during the treatment with depsipeptide, including ST and T wave abnormalities, QTc interval prolongation and cardiac arrhythmias. The Maximum Tolerated Dose (MTD) was determined to be 17 mg/m^2^ on days 1 and 5 every 21 days. Acetylation of histones in patient’s PBMCs was observed confirming inhibition of HDACs by depsipeptide *in vivo* [135, 136].

## CLINICAL EXPERIENCE

### HDAC Inhibitors

The clinical activity of HDAC inhibitors alone is very scarce, although it has been shown that most of them inhibit histone deacetylase activity *in vitro*, synergizing with all-trans retinoic acid (ATRA) in inducing differentiation of myeloid blast cells. Responses to Valproic Acid monotherapy were observed in 8 out of 18 patients (44%) with MDS or sAML/MDS, including 1 partial remission [137]. Valproic acid at serum concentrations of 50-100 μg/ml, combined with ATRA in most of patients, has been used in 58 patients with AML unfit to receive intensive chemotherapy. Response rate was between 5% and 16%, although hematologic improvement and stabilization of the disease was observed [138]. Similar data were reported on 8 refractory or high-risk AML patients, where Valproic acid followed by ATRA induced haematological improvement in 2 patients, while the disease was stable in 5 and progressive in 1 patient [139].

The fatty acid sodium phenylbutyrate sodium PB as a continuous i.v. infusion was administered to 23 patients with AML and MDS, for 7 consecutive days [108]. Prolonged infusions were well tolerated, with only 2 patients on the 21/28 schedule achieving hematological improvement.

A phase 1 trial of orally administered MS-275 was conducted in 38 adults with advanced acute leukemias [129]. The maximum-tolerated dose was 8 mg/m^2^ weekly for 4 weeks every 6 weeks, with DLTs including infections and neurologic toxicity. No responses by classical criteria were seen.

Oral vorinostat (SAHA) at doses 100 to 300 mg twice or thrice daily for 14 days followed by 1-week rest was used in 41 patients with relapsed or refractory leukemias or myelodysplastic syndromes (MDS). Seven patients obtained hematologic improvement, including 2 complete responses and 2 complete responses with incomplete blood count recovery. Increased histone acetylation was observed at all doses and antioxidant gene expression was shown to confer vorinostat resistance [140].

None of these compounds obtained so far FDA approval for treatment of MDS and/or AML, probably because of the low single agent activity shown in clinical trials in AML and MDS patients. Most expectations for these drugs derive from combination studies with hypomethylating agents or other agents with whom synergism has been shown *in vitro*.

On the other end, vorinostat has been approved for the treatment of peripheral T-cell lymphoma and MGCD0103 for the treatment of relapsed-resistant Hodgkin's lymphoma.

### Azacitidine

Although hypomethylating agents have existed for about 30 years, their efficacy has been demonstrated in hematologic malignancies (especially MDS), just in the last 10 years.

The initial dose-finding Phase I and II studies evaluated azacitidine as single agent therapy at 150-200 mg/m^2^/d for 5 days in children with acute leukaemia (14 AML and 22 ALL). Efficacy was high in AML, and the major toxicites reported were nausea, vomiting and diarrhea. Myelotoxicity was also pronounced with nadir of white blood cell counts at 10-14 days [141]. Severe and prolonged myelosuppression was also observed in 154 adults with AML treated at 150-700 mg/m^2^/day for 1-7 days, with rather disappointing clinical responses, ranging from 2 to 15% [142]. Recent trials at lower doses and involving more cycles of therapy have shown the greatest efficacy.

The first randomized controlled trial of low-dose azacitidine versus supportive care in MDS was published in 2002 and led to the FDA approval of the drug. Silverman *et al.* reported on 191 patients with MDS (CALGB 9221 trial), and a low to high IPSS score, randomized to receive azacitidine 75 mg/m^2^/day for 7 days, every 4 weeks, or supportive care [69]. Median times to initial and best response were 64 and 93 days, respectively, with 7% complete (CR), 16% partial response (PR), and 37% haematological improvement (HI) on azacitidine, compared with 5% HI in patients receiving supportive care. Among the 65 patients receiving RBC transfusions at study entry, 45% had an elimination of all transfusions and another 9% had a reduction in transfusions by 50%. In addition, lineage responses for platelets and WBC occurred in 47% and 40% of patients treated with azacitidine. Responses were observed in both low/int-1 as well as int-2/high IPSS score patients and they were associated to longer median time to leukemic transformation or death (21 months for azacitidine versus 13 months for supportive care) [69]. Overall quality of life and social functioning were significantly improved in patients on azacitidine, especially fatigue and psychological state [141].

After the publication in 2000 of the WHO classification [144], MDS with 20-30% of blasts were re-classified as AML. Accordingly, 268 patients treated using azacitidine at 75mg/m^2^/day for 7 days on the CALGB 9221 trial and on 2 previous protocols (CALGB 8421, intravenous infusion and 8921, subcutaneous) were re-analyzed, according also to the IWG response criteria [145]. Response rates were similar in the 3 trials: a median of 3 cycles were required for response, with 90% of patients achieving response by cycle 6. Myelosuppression was a major issue, with 15 days for haemoglobin nadir value, 16 days for platelets and white blood cells and 17 days for neutrophils, but surprisingly there was no increase in the rate of bleeding or infections. Using WHO criteria, 27 AML patients received azacitidine versus 25 patients assigned to observation: 7-16% achieved CR or PR, while 23-32% had HI. Response lasted a median of 7.3 months.

Twenty patients with AML were enrolled in a compassionate azacitidine program between 1996 and 2001 [146]. CR and PR were observed in 20% and 25% of patients, and HI in 15% of patients. Median survival of responders was 15 months, versus 2.5 months for non-responders. Most common toxic effects were febrile neutropenia and pneumonia (6 patients), and 3 patients died due to infection.

In summary, the standard azacitidine doses of 75 mg/m^2^/day for 7 days every 4 weeks, leads to 10-20% CR and PR rates in MDS and AML patients. Treatment is associated with myelosuppression but it results into transfusion independency and prolonged survival in most of the patients. These data have been confirmed by a Phase III, international, multicenter, randomized, prospective trial which included 358 patients with higher-risk MDS patients, FAB-defined as RAEB, RAEB-T, or CMML (10-29% marrow blasts) with an IPSS of Int-2 or High [Fenaux *et al.* ASH 2007, Abstract #817]. This study demonstrated the superiority of azacitidine over three conventional care regimens (CCR; best supportive care, low-dose cytarabine, standard chemotherapy), with a median overall survival of 24.4 months for azacitidine and 15 months for the three CCR. Still, the optimal administration schedule and the possibility to deliver azacitidine on an outpatient basis is an issue. A phase II, multicenter trial [Lyons *et al.* ASH 2007, Abstract #819] is ongoing in MDS, randomizing patients to either azacitidine 75 mg/m^2^/day 5-2-2 (5 days, followed by 2 days stop, and 2 days treatment), azacitidine 50 mg/m^2^/day 5-2-5 (5 days, 2 days stop and 5 days treatment) or azacitidine 75 mg/m^2^/day 5 [for 5 days]. After 6 cycles of azacitidine, patients meeting International Working Group MDS response/improvement criteria defined as CR, PR, stable disease (SD), or HI were eligible to receive an additional 12 cycles. Of 139 randomized patients who had received at least 2 cycles of treatment, HI occurred in 51% of them, with red blood cell transfusion independence. Similar percentages of responses were observed in all 3 alternative azacitidine dosing schedules, with similar safety and efficacy to the FDA-approved regimen.

### Azacitidine Combination Studies

Given the characteristic hypomethylating activity of azacitidine, one of the most attractive combinations is with hystone deacetylase inhibitors. Maslak *et al*. reported on 10 patients (8 AML, two MDS) treated with 7 consecutive daily subcutaneous (s.c.) injections of azacitidine at 75mg/m^2^ followed by 5 days of sodium phenylbutyrate given intravenously (i.v.) at a dose of 200 mg/kg [147]. None of the patients achieved CR, 3 patients achieve PR (2 AML, 1 CMML-2) and 2 other patients had SD; 4 of 5 patients received multiple cycles of this combination. Side effects were mild, but frequent, with over 50% of patients presenting an injection site reaction, somnolence/fatigue, nausea and vomiting. No change in the acetylated forms of histones H3 and H4 were observed following therapy with azacitidine, while, as expected, levels of acetylated histones, in particular H4, increased following phenylbutyrate in patients’ peripheral blood and/or bone marrow mononuclear cells.

The synergistic antileukemia activity of azacitidine and the histone deacetylase inhibitor valproic acid, in the attempt to restore sensitivity to the differentiating effect of all-trans retinoic acid (ATRA) was tested in 49 AML and 4 MDS patients of a median age of 69 years [148]. Patients had refractory or relapsed AML (n=19) or high-risk MDS (n=1), while 33 elderly patients (30 AML, 3 MDS), who refused or were not candidates for chemotherapy, were treated front-line. Three dose levels of valproic acid (50, 62.5 and 75 mg/kg orally days 1-7) were explored, in combination with azacitidine (75 mg/m^2^ sc, days 1-7) and ATRA (45 mg/m^2^ orally, days 3-7 of azacitidine and valproic acid). Median number of courses to first response was 1 (range 1-3), mostly at the valproic acid dose of 50 mg/kg. Cytogenetic response was observed in 36% of patients, CR and CRp occurred in 12 and 3 patients, BM response in 7 patients, with an overall response rate of 42%. Significantly higher response rates were observed in patients reaching higher valproic acid plasma levels.

### Decitabine

In the recent years several studies have been reported using the analogue compound, decitabine. In a phase I study, 48 patients (35 AML, 7 MDS, 1 ALL, 5 CML), mostly with unfavourable karyotype, were treated with low-dose prolonged exposure to decitabine [149]. Dose ranged between 50 and 300 mg/m^2^ per course, with decitabine at 5, 10, 15, or 20 mg/m^2^ i.v. over one hour daily, 5 days/week for 2 consecutive weeks or 15 mg/m^2^ daily for 15 or 20 days. Side effects were tolerable: grade 3 liver dysfunction was observed in 6 patients, febrile episodes were observed in 52% of patients. The best dose schedule was 15 mg/m^2^ daily for 10 days, with a response rate of 83% (4 CR, 1 PR). Higher doses were not associated with increased response rates.

Wijermans *et al.* [78] summarized the results of 177 patients with MDS (23 RA/RARS, 66 RAEB, 65 RAEB-t, 23 CMML), treated in 3 phase II studies. The dose was 40-75 mg/m^2^ continuous infusion in study PCH 91-1 (n=29 patients), while in the PCH 95-11 and PCH 97-19 studies a fixed dose of 15 mg/m^2^ every 8 hours (45 mg/m^2^/d) was given as 3-4-h infusion, 3 days every 6 weeks (median 4 cycles). CR and PR rates were 24% and 10%, HI was observed in 14% of patients, while 18% had progressive disease and there were 12 toxic deaths. Survival was longer in younger patients, with IPSS Int-1 and low risk cytogenetics.

In the U.S., 170 patients with MDS were randomized to receive decitabine at a dose of 15 mg/m^2^ i.v. over 3 hours every 8 hours for 3 days every 6 weeks, or best supportive care [150]. Patients treated with decitabine achieved a 17% overall response rate, including 9% complete responses, while HI was obtained in 13% of patients. Responses lasted for a median of 10.3 months and were associated with transfusion independence. Patients treated with decitabine had a trend toward a longer median time to progression to AML or death (12.1 vs. 7.8 months in the supportive care arm).

Matching patients treated with the 135 mg/m^2^ Decitabine schedule to a hystorical control group of 115 patients with MDS, of similar age, karyotype and an IPSS score Intermediate-1 to High, treated with intensive chemotherapy showed lower early mortality and improved overall survival (22 versus 12 months) for decitabine treatment [151]. In these patients, independent predictors for achieving IWG CR were chronic myelomonocytic leukaemia (CMML) vs MDS, shorter duration of MDS, and no prior MDS therapy, especially when combined.

Similar to azacitidine, the optimal decitabine schedule has not been defined yet. Kantarjian *et al.* randomized 77 patients with MDS and 17 with CMML to 3 different decitabine schedules: 20 mg/m^2^/day i.v. for 5 days, versus 20 mg/m^2^/day s.c. for 5 days, versus 10 mg/m^2^/day i.v. for 10 days. The highest CR rates (39%) and improved epigenetic p15 reactivation were obtained using the 5 day i.v. schedule [79].

One of the major issue is whether hypomethylating treatment should be lifetime, or whether it can be interrupted once response has been achieved. At present, tendency is towards continous mainteinance treatment. The efficacy of retreatment has been shown by Ruter *et al.* who reported on 22 patients with MDS treated with a median of 6 cycles low-dose decitabine, who received decitabine as retreatment at the time of disease recurrence. Retreatment was initiated 11 months after the last course of decitabine: 10 patients (45%) responded (1 CR, 2 PR, 7 HI), while 12 patients did not respond. Second responders to decitabine retreatment were found less frequently in the high-risk IPSS group, compared to non-responders [152].

### Decitabine Combination Studies

Decitabine was combined to valproic acid in 48 patients with AML and 6 MDS [153]. Decitabine was used at a fixed dose of 15 mg/m^2^ i.v., daily for 10 days, concomitantly with escalating doses of valproic acid orally for 10 days. Ten patients obtained CR and 2 CRp, with a median survival of 15.3 months in responders and 4.9 months in nonresponders. Median remission duration was 7.2 months. The 50 mg/kg daily dose of valproic acid was found to be safe and was associated to higher response rate. Transient DNA hypomethylation and global histone H3 and H4 acetylation were induced, and were associated with p15 reactivation. Surprisingly, patients with lower pretreatment levels of p15 methylation had a significantly higher response rate.

To determine an optimal biologic dose (OBD) of decitabine as single agent and then the maximum-tolerated dose (MTD) of valproic acid combined with decitabine, 25 patients with AML (13 untreated, 13 relapsed) of a median age of 70 years were treated in a phase I trial [154]. Fourteen patients received decitabine alone and the OBD was 20 mg/m^2^/day i.v. for 10 days. Eleven patients received decitabine 20 mg/m^2^/day for 10 days plus dose-escalating valproic acid (days 5-21). Of 21 assessable patients, 11 (52%) responded: 4 with morphologic and cytogenetic CR; 4 with incomplete CR, and three with PR. In untreated AML, 4 of 9 assessable patients achieved CR. Clinical responses appeared similar for decitabine alone or with valproic acid. Dose-limiting encephalopathy occurred in 2 of 2 patients at valproic acid 25 mg/kg/day and 1 of 6 patients at 20 mg/kg/day dose.

Low-dose decitabine (5 mg/m^2^/day i.v., 5 days/week for 2 weeks) was combined to 600 mg/day imatinib in a phase II study in patients with CML in accelerated and myeloid blastic phase [155]. After 91 cycles in 28 patients (25 with imatinib resistance; 18 in accelerated, 10 in blastic phase), CR were observed in 9 (32%), PR in 1 (4%), and HI in 2 (7%) patients. Major and minor cytogenetic responses were observed in 5 (18%) and 3 (11%) patients. The hematologic response rate was higher in patients without BCR-ABL kinase mutations (10 of 19) than in those with mutations (1 of 7). Median duration of hematologic response was 18 weeks, with myelosuppression as the major adverse effect resulting in neutropenic fever in 9 patients (32%).

### Prognostic Factors Associated with Outcome

Although CR rates in AML and MDS range between 10-20%, the major advantages of epigenetic therapy is hematologic improvement, transfusion independency, and delayed transformation into AML, which results into prolonged survival, especially in MDS patients. Since this is a biologic approach, it remains to define which are the molecular factors associated to best responses, to identify patients who may best profit from treatment. Several studies have shown reactivation of silenced genes after hypomethylating therapy *in vitro*, but this effect is still matter of debate *in vivo*.

Gore *et al*. demonstrated a significant decrease of *p15* methylation in MDS and AML patients treated with low-dose azacitidine and the histone deacetylase inhibitor sodium phenylbutyrate. Reexpression of p15 protein was increased and correlated with clinical response. Surprisingly, azacitidine alone was sufficient to increase the overall acetylation level of histones H3 and H4, which was further increased upon phenylbutyrate treatment [156].

Using azacitidine and valproic acid, Soriano *et al.* showed using a bisulfite pyrosequencing assay, that global DNA methylation, was not associated to response, but significantly decreased by day 7, and returned towards baseline by day 0 of the next cycle [148]. Although a modest but significant increment on p15 and p21 mRNA expression was detected, this was not associated with clinical response. Similarly, valproic acid dose level and histone acetylation did not correlate to response.

In the same way, bisulphite genomic sequencing of the p15 promoter showed 12.2% pre-treatment methylation in 14 of 17 patients treated with Azacitidine. p15 methylation was reduced by 6.8% in 8 of 17 patients, but this did not correlate with response. Significantly lower baseline methylation occurred in responders (9.8% versus 16.2% in non-responders), while hypermethylation over 24% and absent p15 mRNA expression were associated to lack of response. Cell death with reduced bone marrow cellularity and increased apoptosis, rather than p15 demethylation better correlated with response [44].

These data show that since hypomethylation is induced both in normal and neoplastic cells, the analysis of global methylation dynamics could be considered as a marker of the biological activity of the drug, but it is not necessarily related to clinical activity. Specific gene methylation studies, other than p15, may identify more reliable markers of response. Accordingly, in patients treated with decitabine and valproic acid, drug-induced re-expression of estrogen receptor was associated with clinical response [154]. ER promoter demethylation, global DNA hypomethylation, depletion of DNA methyltransferase enzyme, and histone hyperacetylation were also observed.

It is also possible that the clinical activities of both azacitidine and valproic acid are only partially related to the inhibition of DNA methylation and of hystone deacetylation, and that other mechanisms may be prevalent.

Cytogenetic abnormalities including complex karyotype and chromosome 7 anomalies have been associated to increased response rates to hypomethylating therapy. Of 34 patients with MDS or AML, treated with azacitidine, 5 of 7 complete responders had chromosome 7 abnormalities [44]. On the contrary, Kantarjian *et al.* showed that independent adverse prognostic factors for survival for 115 patients with high-risk MDS were chromosome 5 and/or 7 abnormalities, older age, and prior MDS therapy (excluding growth factors) [157]. The National Cancer Research Institute trial AML14 comparing low-dose cytarabine and hydroxyurea showed no benefit for patients with MDS and AML and adverse cytogenetics [158].

When looking at morphology, patients with CMML were reported to have increased reponse rates. decitabine at 100 mg/m^2^ per course every 4 weeks was administered for a median of 9 courses to 19 adults with CMML. Eleven patients (58%) achieved CR and 2 had HI, for an overall response rate of 69% according to the modified IWG criteria [159].

## CONCLUDING REMARKS AND FUTURE DIRECTIONS

Azacitidine and decitabine, two cytidine analogues, were the first demethylating agents entered in clinical trials. First used at higher doses, as cytotoxic agents, for the treatment of AML, these demethylating agents gave definetively more satisfying results when used at low dose schedule for the treatment of MDS. When compared to supportive care, Azacitidine and Decitabine showed high overall response rate, reduced transfusion requirement, improved quality of life and reduced risk of leukemic transformation in MDS patients. In particular, Azacitidine significantly prolonged survival in MDS patients when compared to conventional care regimens in the AZA-001 Phase III randomized prospective trial [Fenaux *et al.* ASH 2007, Abstract #817], while a survival advantage was described for decitabine over intensive chemotherapy in high risk MDS patients compared with matched historical controls [160]. A molecular biomarker of responsivity to demethylating treatment has still to be identified, since no stable correlation has been observed between response to azanucleotides and promoter methylation of single genes, as p15. New oral DNMT inhibitors, as oral azacitidine and zebularine, are under investigation.

Valproic acid and Sodium phenylbutirrate, the first HDAC inhibitors studied, induce terminal differentiation of leukemic blasts *in vitro* and showed scarce activity when used as single agents *in vivo.* Since these drugs were shown to synergize with DNMTs inhibitors in inducing re-expression of silenced gene, combination studies with both inhibitors are undergoing, although it is not clear yet whether HDAC inhibitors add to the efficacy of hypomethylating agents. New more effective and well tolerated HDAC inhibitors, including depsipeptide, SAHA, LBH589, PDX101, MS-275, CI-994 and MGCD0103, entered in clinical trials alone or in combination. These compounds will hopefully hold *in vivo* the premises of the epigenetic reactivation often reported *in vitro*, with a more acceptable toxicity profile.

## Figures and Tables

**Table 1:** Genes Hypermethylated in MDS and AML

Pathway	Gene	MDS	AML
Cell cycle	CDKN2B (p15)	50-80%	30-71%
Signal transducers	SOCS1	11 - 31%	0-60%
	RASSF1A	9%	-
	SHP-1	-	11%
Angiogenesis	E-cadherin (CDH1) Thrombospondin	30-70%	13-64%
		6%	-
Apoptosis	DAP-kinase	7-17%	3-27%
	p73	-	10 -37%
DNA repair	MGMT	2%	5%
	BRCA1	-	38%
	MLH1	-	4%
Hormones and H-receptors	Calcitonin (CALCA) Estrogen receptor Retinoic acid receptor β2	83%	71 – 95%
		19%	40 – 69%
		-	18 - 43%
Detoxification	GSTP1	-	18%
Others	HIC1	32%	10%

**Table 2:** HDAC Inhibitory Activities of Distinct Drugs

HDAC inhibitor class	Drug	HDAC1 IC50 *(µM)*	HDAC2 IC50 *(µM)*	HDAC3 IC50 *(µM)*	HDAC4 IC50 *(µM)*	HDAC6 IC50 *(µM)*	HDAC8 IC50 *(µM)*
Short chain fatty acids	Valproic acid [103]	700	800	1000	1500	>20000	*n.a*
Hydroxamic acids	SAHA [88]	0.021	*n.a*	0.037	*n.a*	0.025	1.2
	LAQ824/LBH589 [88]	0.0018	*n.a*	0.0037	*n.a*	0.015	0.14
Benzamides	MS-275 [88]	0.18	*n.a*	0.74	*n.a*	>100	44.9
	CI-994 [88]	0.41	*n.a*	0.75	*n.a*	>100	>100
	MGCD0103 [88]	0.082	*n.a*	0.62	*n.a*	>30	>25
Cyclic peptides	Despipepdide [134]	0.036	0.047	*n.a*	0.51	14	*n.a*

Concentrations that inhibits 50% (IC50) of the activity for each HDAC isoenzyme of distinct HDAC inhibitors. Comparison is only indicative because different HDAC assays were used in the reported studies [88, 103, 134]. The IC50 was not available *(n.a.)* for all the HDAC isoenzymes.
